# Rumination in the goat (*Capra hircus*) is a circadian rhythm synchronized by feeding time

**DOI:** 10.3389/fphys.2026.1818222

**Published:** 2026-05-19

**Authors:** Mohammed El Mehdi M’Hani, Younes Beniaich, Rachid El Moussaouiti, Hicham Farsi, Mohammed Piro, Mohamed Rachid Achaâban, Paul Pévet, Etienne Challet, Khalid El Allali

**Affiliations:** 1 Comparative Anatomy Unit, Department of Biological and Pharmaceutical Veterinary Sciences, Hassan II Agronomy and Veterinary Medicine Institute, Rabat, Morocco; 2 Medicine and Surgical Unit of Domestic Animals, Department of Medicine, Surgery and Reproduction, Hassan II Agronomy and Veterinary Medicine Institute, Rabat, Morocco; 3 Institute of Cellular and Integrative Neurosciences, Centre national de la recherche scientifique (CNRS) and University of Strasbourg, Strasbourg, France

**Keywords:** circadian clock, desert goat, entrainment, feeding, rumination rhythm

## Abstract

**Introduction:**

Rumination is usually considered to be a daily rhythmic process that is not dependent on food intake. Although this rhythm is described as circadian in the literature, to our knowledge, no experimental study has yet demonstrated its circadian origin. The objective of the present study was to explore the circadian control of the rumination rhythm in the goat (Capra hircus) and its possible entrainment by the timing of food availability.

**Methods:**

Rumination was continuously recorded in bucks housed under constant darkness (DD) and constant ambient temperature (CTa). During Experiment 1 (n=6), food was provided once daily at 10h in Stage 1, four times a day (00h, 06h, 12h, and 18h) in Stage 2, ad libitum in Stage 3, and finally in Stage 4, bucks were food-deprived for four consecutive days. Experiment 2 was designed to investigate the effect of time-shifting of food distribution on the rumination rhythm under constant conditions. Food was given once a day, at 10h in stage 1, then shifted to 22h in stage 2 and returned to 10h in stage 3.

**Results:**

In Stage 1 and 2 of Experiment 1, rumination exhibited a clear rhythmic profile with a period not significant from 24.0 h. In Stage 3, the rhythm exhibited a progressive daily phase shift typical of a free-running state with a circadian period of 24.9 ± 0.1 h. During Stage 4, food deprivation did not abolish rumination or its rhythmicity, though it did alter the free-running period. Experiment 2 showed that the inversion time of the food distribution immediately induced a shift in rumination rhythm, the acrophases were delayed from 02h56 ± 28min (stage 1) to 14h55 ± 8min (stage 2). The return to amorning feeding in stage 3 has led to a phase advance in rumination rhythm, the acrophases being advanced from 14h55 ± 8min (stage 2) to 02h07 ± 13min (stage 3).

**Discussion:**

This study provides the first evidence supporting a circadian origin of the rumination rhythm in goat. The results also clearly showed that feeding time was able to synchronize the rumination rhythm. Nevertheless, additional research is required in order to elucidate the complete neurobiological mechanisms underlying the circadian control of rumination in the goat.

## 1 Introduction

Rumination is a physiological phenomenon characteristic of a large number of animals belonging to the suborder Ruminantia. It is the process by which fibrous digesta is regurgitated from the rumen into the oral cavity where it is re-masticated, re-ensalivated, formed into a bolus and then swallowed back into the rumen (Welch, 1982; Beauchemin, 1991, Beauchemin, 2018). This physiological process is an adaptation in ruminants that allows them to consume large amounts of fiber-rich food in a relatively short period of time, followed by rumination during resting periods (Reinhardt and Faris, 2014). Beyond its primary digestive function, rumination shows a particular temporal distribution. Indeed, several studies have reported that rumination has a rhythmic profile, occurring predominantly at night in diurnal ruminants (Gordon, 1958a; Hancock, 1954; Gordon and McAllister, 1970; Bell and Lawn, 1957a; Oshiro, 1985; Farsi, 2020).

Although the physiological aspects of rumination have been extensively studied, the precise cerebral mechanisms that control this behaviour remain unknown. A pioneer study by Bell and Lawn (1957a) showed that bilateral lesion of the orbito-frontal lobes in goats disrupted normal rumination, suggesting a cortical involvement in its regulation. Subsequent research has suggested the possible involvement of the peptide gastrin-17 (Honde and Bueno, 1984) and dopamine, possibly through pathways involving alpha-2 adrenergic receptors (Buéno et al., 1983), in the central control of rumination. It has also been suggested that the hypothalamus may be involved, given its established role in modulating reticulorumen contractions, which are essential for rumination (Kania, 1994). However, these results remain largely speculative and do not allow any definitive conclusions about the cerebral control of rumination. While the cerebral mechanisms involved in rumination remain largely speculative, the evidence for central nervous system implication raises the question of whether this behaviour may be under the control of the central circadian clock located in the suprachiasmatic nucleus of the hypothalamus (SCN).

Several studies have attempted to identify the factors that regulate rumination and its rhythmicity. Early research suggested that rumination is primarily initiated by feeding and that its rhythm is determined by the physicochemical properties of the ingested food (Pearce, 1965a; Welch and Smith, 1971). Alternatively, other studies have suggested that rumination rhythm may be more dependent on light-dark (LD) cycles, or at least partially influenced by both photoperiod and feeding (Gordon and McAllister, 1970; Bell and Lawn, 1957b). Later, evidence has emerged that rumination can persist with a rhythmic profile even in the absence of feeding (Oshiro et al., 1992). In a recent study, M’Hani et al. (2024) provided compelling evidence that rumination and its rhythmicity are not food dependent. Although prolonged food deprivation significantly reduced the total daily rumination time, the behaviour persisted with a robust rhythmic profile similar to that observed under normal feeding conditions. A common feature of these studies is the characteristic pattern of the rumination rhythm, suggesting that it may be controlled by circadian clock(s).

To be classified as circadian, a rhythm should be generated by a circadian clock, such as the master clock located in the SCN. This implies that the rhythm should persist under constant environmental conditions (continuous light (LL) or darkness (DD), constant ambient temperature (CTa), and the absence of other temporal cues such as food availability or social interactions) with a period close to but different from 24 hours (Boissin and Canguilhem, 1998; Dibner et al., 2010; Refinetti, 2006). Although several studies, including the pioneer work by Gordon and McAllister (1970), have described the rhythm of rumination as circadian, definitive experimental evidence to support this classification remains to be provided. In this context, the present study aims to investigate whether rumination in goats is a true circadian rhythm by studying the behaviour in the absence of temporal cues. Subsequently, the study has been extended to explore more deeply the relationship between the rumination rhythm and the timing of food availability.

## 2 Experimental procedures

### 2.1 Animals

The study was conducted on 16 healthy adult male Moroccan black goats (*Capra hircus*), weighing 31 ± 1.5 kg and aged approximately 3.5 years, originating from the south of Morocco. The animals were housed in groups under indoor conditions in an experimental room at the Hassan II Institute of Agronomy and Veterinary Medicine in Rabat (latitude: 34°01′N, longitude: 6°50′W). The bucks were not restrained and were able to move freely. Water was a*d libitum*.

In order to reduce the number of animals and refine their use, the research was conducted using male goats that were intended for dissection by veterinary students and for anatomy teaching in the veterinary school of the Hassan II Institute of Agronomy and Veterinary Medicine. Animals were randomly attributed on two groups. Group 1 consisting of 6 bucks was used in experiment 1; while Group 2 formed by 10 bucks was used for experiment 2. All experimental procedures were reviewed and approved by the Hassan II Institute of Agronomy and Veterinary Medicine Local Ethics Committee (Committee of Ethics for Animal Science and Veterinary Public Health: CESASPV) under two separate approval numbers: CESASPV_2024_A05 and CESASPV_2024_A07.

### 2.2 Experimental design

The experiments were conducted in a closed, isothermal experimental room where the ambient temperature, humidity, and light-dark cycle were artificially controlled. The relative humidity of the air was around 23%. Food consisted of a complete compound feed (Maraa^®^, Alf-Sahel, Morocco) and hay. The compound feed was formulated with cereals (maize, barley), fibers (wheat bran), oilseed cake (soya, sunflower) and a mineral and vitamin premix (**Table 1**). The daily ration comprises 400 g of complete compound feed and 2 kg of hay per animal. This ration remained constant across all stages of both experiments; only the feeding schedule was changed.

**Table 1:** A summary of the chemical composition of the used food.

Characteristics	Components
MINIMUM (%)	Crude protein 14%
	Crude fat 2%
	Phosphorous 0,3%
	Calcium 0,6%
MAXIMUM (%)	Minerals 10%
	Crude fibre 12%
	Water 13%
VITAMINS (International Units/1KG)	Vitamin A 1000 IU/KG
	Vitamin D 200 IU/KG
	Vitamin E 20IU/KG

EXPERIMENT 1: was conducted on Group 1 to investigate the circadian origin of the rumination rhythm. Both *ad libitum*, 4-meals feeding and subsequent food deprivation were used to eliminate food availability as a potential temporal cue influencing the rumination rhythm. The experiment consisted of five stages:

Adaptation stage (5 days): this was an adaptation period to indoor conditions with an artificial LD cycle (12L-12D) and uncontrolled Ta (18.00-24 °C). Food was given once daily at 10h am (GMT + 1) which correspond to the normal practice feeding used in these bucks before the experiment. The objective of this stage was to replicate the preceding outdoor natural conditions in a controlled experimental room before subjecting goats to specific experimental conditions.

Stage 1 (14 days): Animals were placed under conditions of total darkness (DD) and constant ambient temperature (CTa) of 21 ± 0.5 °C. Food regime was as in the previous stage, once a day at 10h am (GMT + 1).

Stage 2 (8 days): Animals were under conditions of DD and CTa of 21 ± 0.5 °C. Food was given 4 times a day at 00h, 06h, 12h and 18h (GMT + 1), this was to test whether 4 food distributions a day with a 6 hours interval preclude any entrainment of the circadian rhythm (El Allali et al., 2013; Piccione et al., 2002).

Stage 3 (21 days): DD + CTa of 21 ± 0.5 °C. Food was available ad libitum to avoid any entrainment of circadian rhythms (Farsi et al., 2020b).

Stage 4 (4 days): DD + CTa of 21 ± 0.5 °C with no access to food but with water *ad libitum*. It should be noted that the variety of Moroccan black goat used in this experiment is well adapted to the arid desert environment and is known for its ability to endure prolonged periods of food and water deprivation (Hossaini-Hilali and Benlamlih, 1995).

EXPERIMENT 2: was conducted on group 2 to assess whether, in the absence of LD and Ta cycles, shifting the time of food distribution could synchronize the rumination rhythm. The experiment was conducted in four stages as follows:

Adaptation stage (5 days): bucks were adapted to indoor conditions with an artificial LD cycle (12L-12D) and uncontrolled Ta (18.00-25 °C). Food was given once daily at 10h (GMT + 1).

Stage 1 (09 days): Animals were placed under conditions of total darkness (DD) and constant ambient temperature (CTa) of 20.5 ± 0.5 °C. Food was given once daily at 10h (GMT + 1) during the subjective day.

Stage 2 (21 days): Animals were under similar DD and CTa. However, food distribution was delayed by 12 hours. Food was given once daily at 22h (GMT + 1) during the subjective night.

Stage 3 (09 days): DD and CTa conditions have been maintained but feeding time has been returned to the previous conditions of stage 1 with single food distribution per day at 10h (GMT + 1) during the subjective day.

### 2.3 Rumination recording

Animals were monitored by video-recording using two high-resolution cameras equipped with infrared emitters (Dahua^®^ HACHFW1200RM cameras made by Dahua Technology Co., Ltd in Zhejiang, China). The recorded videos were stored into a Digital Video Recorder (Dahua DVR (DHIHCVR5108H-S2, Dahua Technology Co., Ltd, Zhejiang, China) with the capacity to hold 4 Terabytes for subsequent sequences analysis of rumination.

Based on the recorded video footage, rumination was scored for all bucks throughout the experimental periods in the two experiments. Observations were made at 30-second intervals, with a score of ‘1’ given when rumination was observed and a score of ‘0’ given when rumination was absent. Absence of rumination included periods when animals were chewing during feeding or showed no chewing activity. In cases where a 30-second epoch included two different behaviours, only the predominant behaviour (lasting more than 15 seconds) was considered (M’Hani et al., 2024). Rumination duration was then calculated and expressed in minutes per day.

### 2.4 Data analysis

Total rumination time for each stage was presented as mean ± standard error of the mean (SEM) and plotted using GraphPad Prism software (GraphPad Prism 8.0.2). Total rumination time (mean ± SEM) was calculated by pooling the rumination duration data from each animal for each 24-h period, averaging over the days of each stage, and then averaging over all animals in the experiment.

Rumination data were presented also as individual and mean ruminograms using Actogram Plotter^®^ software (Refinetti R, Circadian Rhythm Laboratory, University of South Carolina, http://www.circadian.org/softwar.html). A 5 min interval time-set was used to plot ruminograms. Scores of 0 to 5 representing a rumination lasting respectively from 0 to 5 min in each 5 min interval time set have been assigned to facilitate the design of ruminograms.

Rumination rhythm parameters (period, acrophase, robustness, amplitude and mesor) were calculated by non-linear least squares regression using Acro^®^ and Cosinor^®^ softwares (Refinetti R, Circadian Rhythm Laboratory, University of South Carolina, http://www.circadian.org/softwar.html).

Comparisons of total rumination time and rhythm parameters between the different stages of each experiment were performed using one-way ANOVA with repeated measures, followed by a Holm-Sidak *post hoc* test. A one-sample t-test was also performed to assess whether the periods were significantly greater or less than 24 h. Statistical significance for all tests was set at P ≤ 0.05.

## 3 Results

### 3.1 Experiment 1

**Figure 1:**
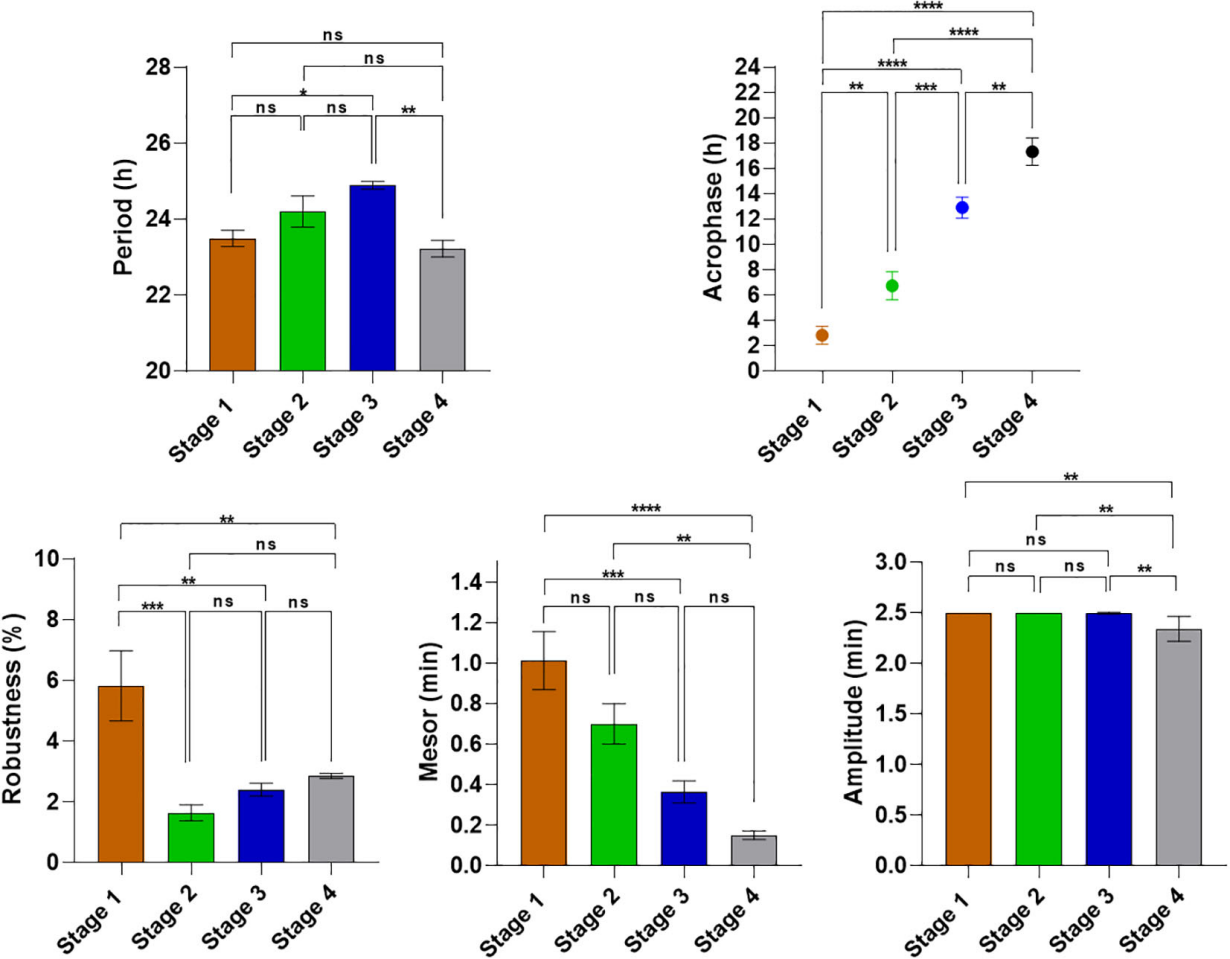
Comparison of rumination rhythm parameters between the different stages of the experiment 1. Comparisons between the different stages were performed using the One-way ANOVA with repeated measures. ****p < 0.0001 (Extremely significant differences). ***p < 0.001 (Very highly significant differences). **p < 0.001 (highly significant differences). *p < 0.05 (significant differences). ns: p > 0.05 (non-significant differences).

**Figure 2:**
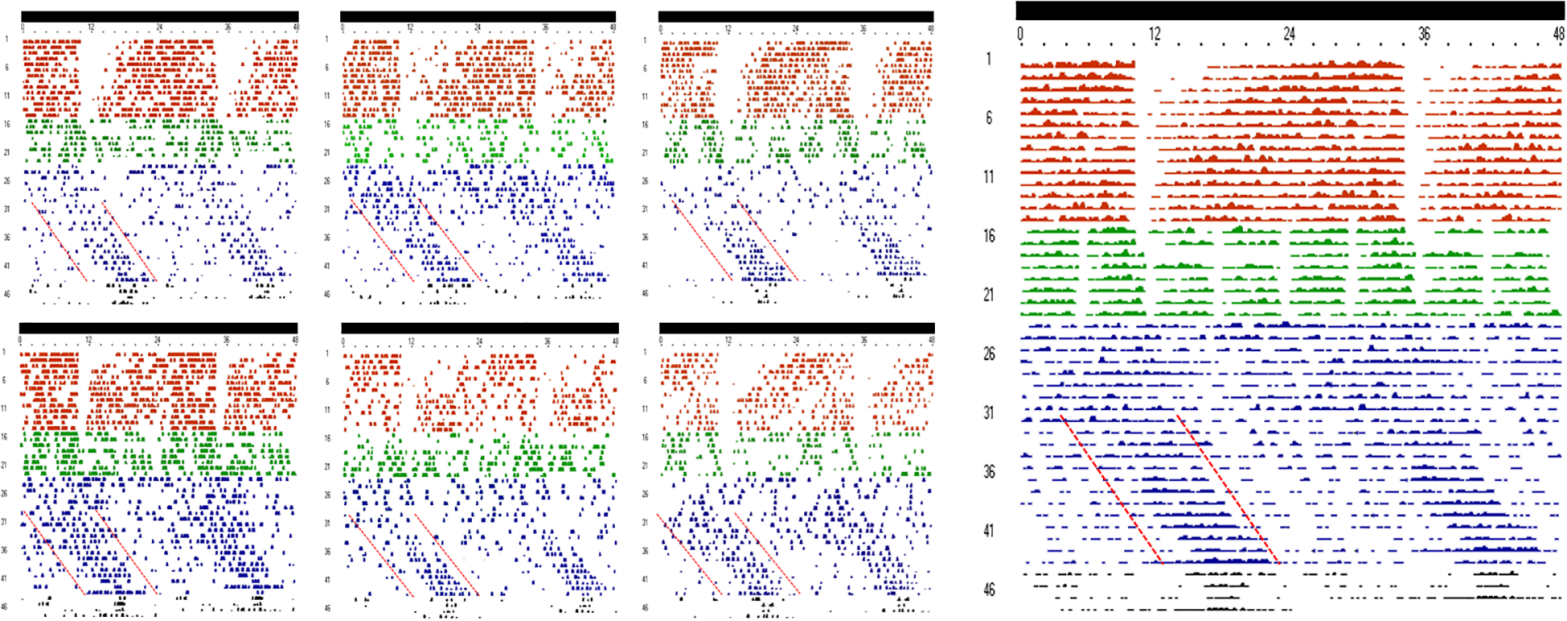
Individual and mean double plotted ruminogram of rumination rhythm during stages 1,2, 3 and 4 of the experiment 1. Each line corresponds to 24 h of rumination starting at 00:00 h and ending at 24:00 h of the following day. The coloured lines and points indicate the existence of rumination. Brown for Stage 1, green for Stage 2, blue for Stage 3, and black for Stage 4. Scores of 1 to 5 for (see methods section) induce an increase in the thickness of the line; while white or on-line vacuum denote of no rumination (Scores of 0). Long black bar indicates total darkness (DD).

Under the experimental conditions of Stage 1, Cosinor regression showed a clear rhythmic profile of rumination with a period of 23.5 ± 0.2h which is not statistically different from 24.0 h (p=0.0514). The robustness was of 5.8 ± 1.1% and the acrophase occurred during subjective night at 02h50 ± 42 min. The mesor and amplitude were 1 ± 0.14 and 2.5 ± 0.0 min, respectively (**Figures 1**, **2**). When food was distributed four times a day (00h am, 06h am, 12h pm and 18h pm) in stage 2, rumination exhibited an irregular rhythm (**Figure 2**) with a significantly reduced robustness (F _(3.20)_ = 9; p=0.0005), decreasing from 5.8 ± 1.1% in stage 1 to 1.65 ± 0.26% in stage 2. The acrophase shifted from the subjective night to the subjective day (F _(3.20)_ = 44.64; p=0.0099), occurring at 6 h 43 ± 65 min. However, no significant changes were observed in either the mesor (F _(3.20)_ = 16.91; p=0.06) or the amplitude (F _(3.20)_ = 9.34; p>0.99) when comparing stages 1 and 2. The period was not significantly different from 24.0 h (24.2 ± 0.4 and, p=0.644). During stage 3, environmental conditions were strictly constant (DD, CTa, and ad libitum feeding), thereby eliminating all potential temporal cues. Under these conditions, the rhythmicity of rumination persisted with a robustness of 2.4 ± 0.2%. The one-sample t-test showed that the period in stage 3, with a mean of 24.9 ± 0.1 h, was significantly longer than 24.0 h (p<0.0001). Double-plotted ruminograms (**Figure 2**) clearly showed a gradual daily shift in rumination during stage 3, indicating that the rhythm of rumination was in a free-running state. This free-run was confirmed by a significant delay in the acrophase, which shifted from 6 h 43 ± 65 min during stage 2 to 12 h 54 ± 49 min during stage 3 (F _(3.20)_ = 44.64; p=0.0005). All together, these results provide clear evidence for a circadian origin of the rumination rhythm. In stage 4, the bucks were maintained under constant conditions (DD and CTa) without access to food. Despite the food deprivation, rumination rhythm persisted with a period of 23.2 ± 0.2 h (significantly different from 24.0 h, p=0.016) and a robustness of 2.85 ± 0.08%. The acrophase was also significantly delayed during this stage (F _(3.20)_ = 44.64; p=0.0095) to occur at 17 h 19 ± 64 min.

**Figure 3:**
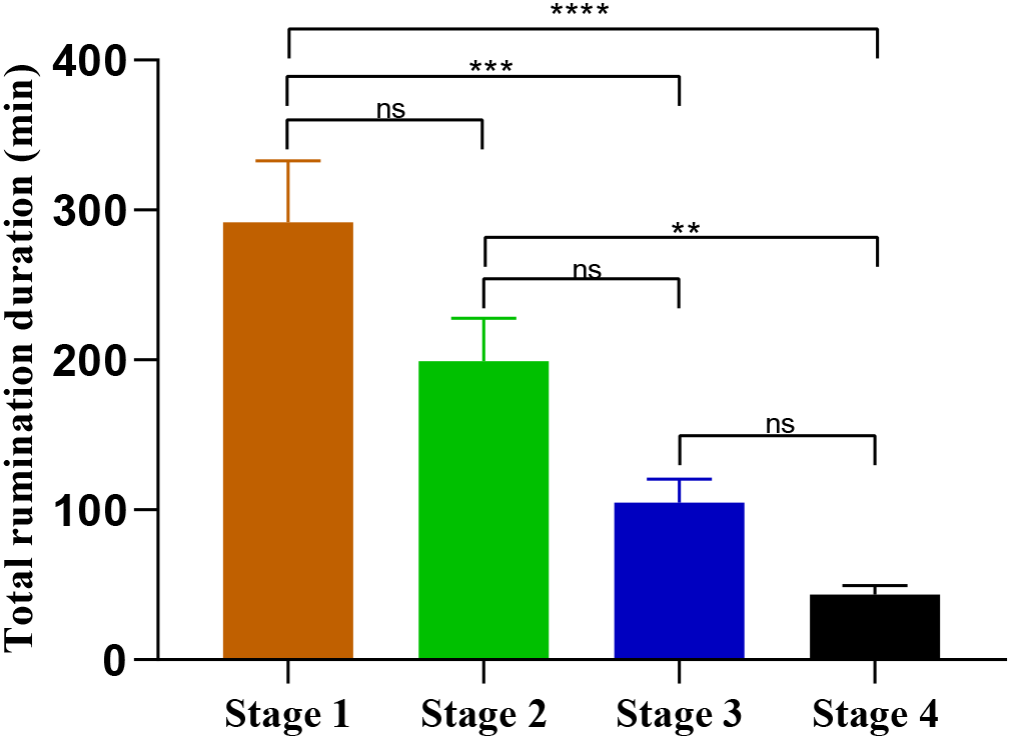
Total rumination duration (mean ± SEM) per day during stages 1, 2, 3 and 4 of the experiment 1. For each animal, data of rumination duration were pooled for 24-h and then averaged over the days of each stage thereafter averaged for the 6 animals. Comparisons between the different stages were performed using the One-way ANOVA with repeated measures. ****p < 0.0001 (Extremely significant differences). ***p < 0.001 (Very highly significant differences). **p < 0.001 (highly significant differences), ns, p > 0.05 (non-significant differences).

In Stage 1, the total daily rumination duration was 291.6 ± 41 min which represent 20.2% of the 24h daily duration. Following the transition to four food distributions per day in stage 2, this parameter decreased to 199 ± 28 minutes (13.8% of 24h). Under ad libitum feeding conditions of stage 3, total rumination duration decreased further to 105 ± 15 minutes (7.3% of 24h). The lowest total rumination duration was observed during the food deprivation stage, with an average of 43 ± 6 min (3% of 24 h) (**Figure 3**). A one-way ANOVA revealed a significant decrease in the total rumination duration across the four stages of the experiment as they progressed. Stage 1 showed the highest mean duration. Although the decrease observed in Stage 2 was clear, nevertheless it remained not statistically significant (F _(3.20)_ = 16.9; p=0.06). In contrast, the decreases observed in Stages 3 (F _(3.20)_ = 16.9; p=0.0004) and 4 (F _(3.20)_ = 16.9; p<0.0001) were statistically significant compared to stage 1. Indeed, rumination duration in stage 4 was significantly lower compared to stage 2 (F _(3.20)_ = 16.9; p=0.0019). Overall, the data indicate a marked decrease in rumination behaviour over the experimental stages.

### 3.2 Experiment 2

**Figure 4:**
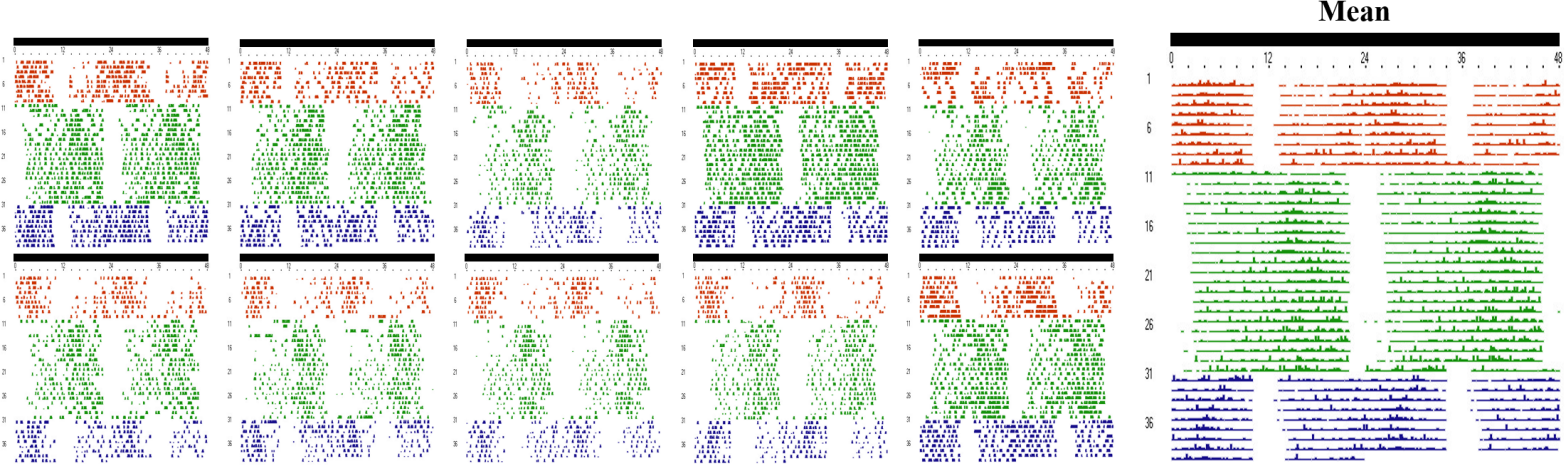
Individual and mean double plotted ruminogram of rumination rhythm during stages 1,2 and 3 of the experiment 2. Each line corresponds to 24 h of rumination starting at 00:00 h and ending at 24:00 h of the following day. The coloured lines and points indicate the existence of rumination. Brown for Stage 1, green for Stage 2 and blue for Stage 3. Scores of 1 to 5 for (see methods section) induce an increase in the thickness of the line; while white or on-line vacuum denote of no rumination (Scores of 0). Long black bar indicates total darkness (DD).

During experiment 2, rumination exhibited a clear rhythmic profile throughout all stages (**Figure 4**). The one-sample t-test indicated that the rhythm period across the three stages did not differ from 24.0 h (p = 0.374), with periods of 23.6 ± 0.2 h in stage 1, 24.0 ± 0.02 h in stage 2, and 24.0 ± 0.1 h in stage 3. This demonstrates that the timing of one food distribution a day is able to synchronize the rumination rhythm to a 24-hour period.

**Figure 5:**
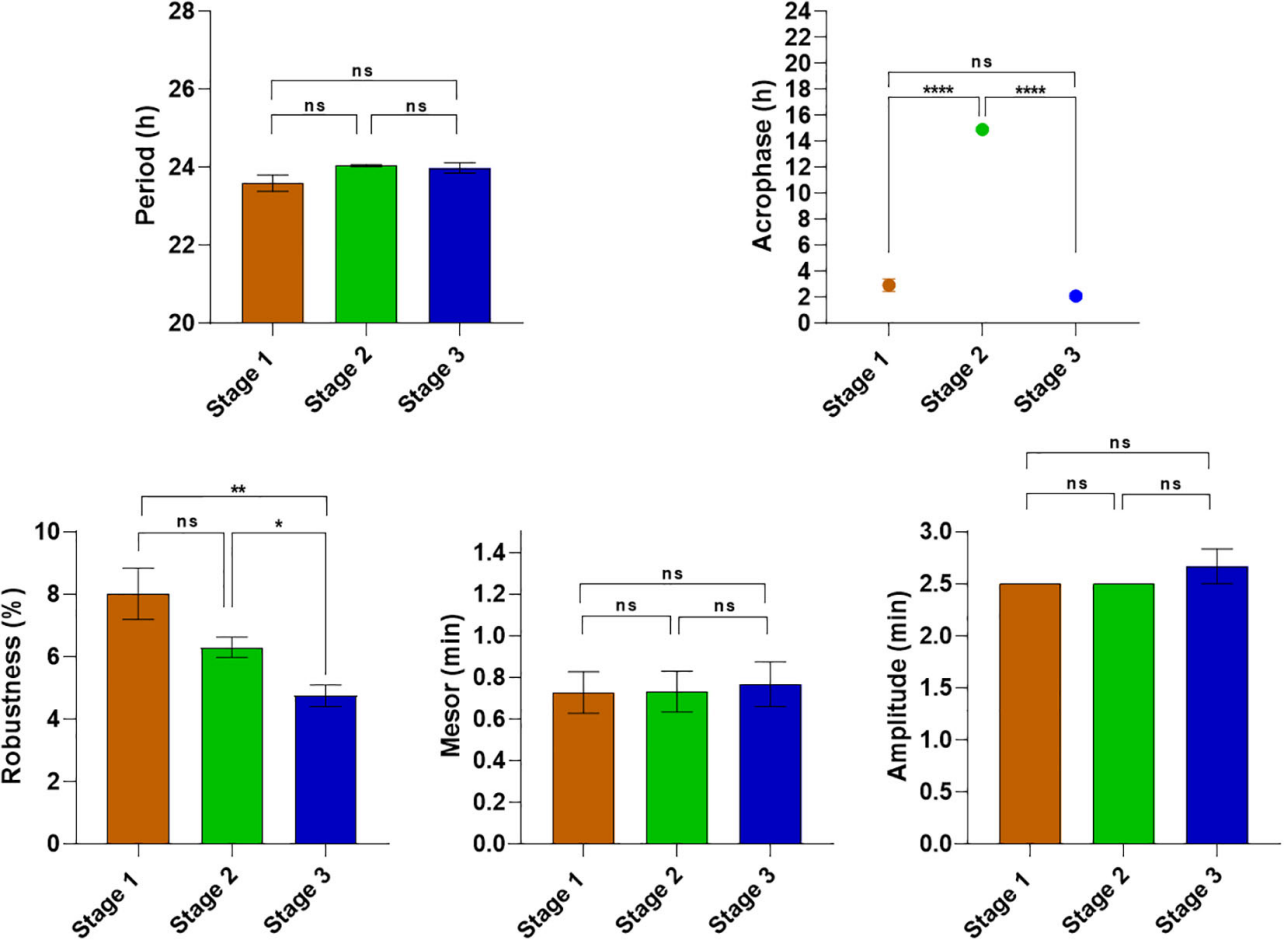
Comparison of rumination rhythm parameters between the different stages of the experiment 2. Comparisons between the different stages were performed using the One-way ANOVA with repeated measures. ****p < 0.0001 (Extremely significant differences). **p < 0.001 (highly significant differences). *p < 0.05 (significant differences). ns, p > 0.05 (non-significant differences).

A one-way ANOVA with repeated measures was conducted to compare the parameters of the rumination rhythm across the different stages (**Figure 5**). The results demonstrated the absence of any significant changes in the period, mesor, and amplitude across stages. However, the robustness of the rhythm showed a significant decrease across the different stages. Differences are significant between stage 2 (6.3 ± 0.33%) and stage 3 (4.75 ± 0.35%) (F _(2,27)_ = 6.837; p = 0.0186), and also between stage 1 (8 ± 0.8%) and stage 3 (F _(2,27)_ = 6.837; p = 0.005). Cosinor regression showed that rumination acrophase during stage 1 (food at 10h a.m) occurred during the subjective night at 02h 56min ± 28min; while a 12-hour shift in the time of food distribution in stage 2 (10h p.m) has led to an inversion of rumination acrophase to occur at 14h 55 ± 08min, during the subjective day (F _(2.27)_ = 529.5; p<0.0001). The return to a daytime feeding at 10h a.m. during stage 3 resulted in a complete return to a subjective night-time acrophase, occurring at 02h 07 ± 13min (F _(2.27)_ = 529.5; p<0.0001). The return was complete, as confirmed by the absence of a statistically significant differences of acrophase between stages 1 and 3 (F _(2.27)_ = 529.5; p=0.068). The results show that a 12-hour shift in the time of food distribution leads to an immediate parallel shift in the acrophase of the rumination rhythm. The phase shift in the rumination rhythm subsequent to the changes in the timing of food distribution was observed to be immediate without any gradual or progressive shift (**Figure 4**).

**Figure 6:**
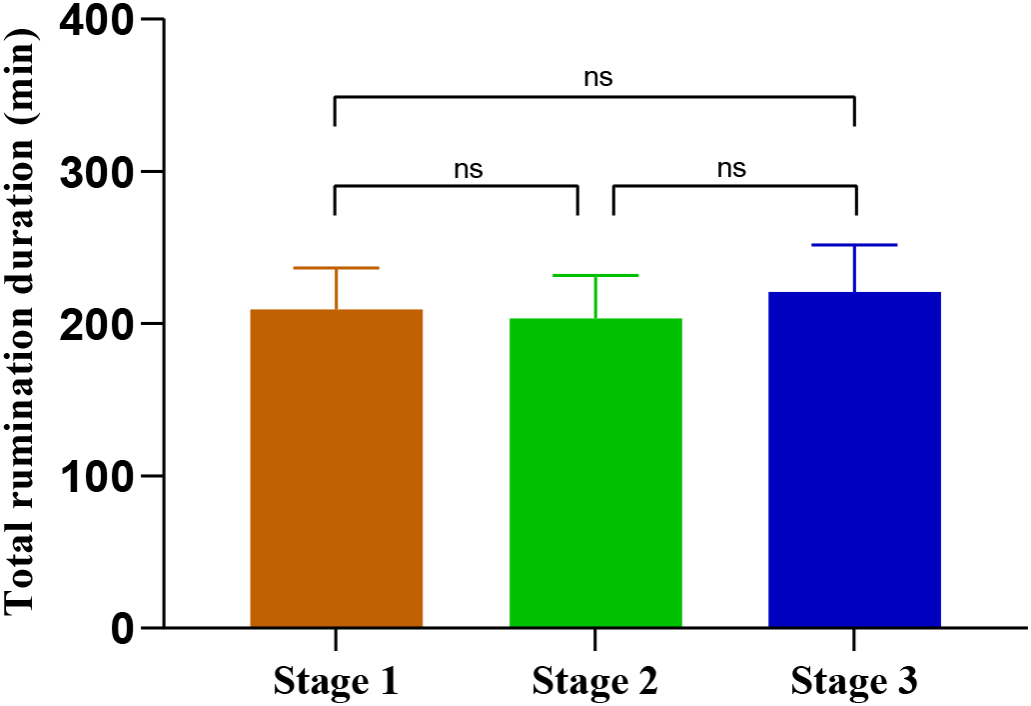
Total rumination duration (Mean ± SEM) per day during stages 1, 2 and 3 of the experiment 2. For each animal, data of rumination duration were pooled for 24-h and then averaged over the days of each stage thereafter averaged for the 10 animals. Comparisons between the different stages were performed using the One-way ANOVA with repeated measures. ns, p > 0.05 (non-significant differences).

The one-way ANOVA with repeated measures revealed no significant differences in total daily rumination duration among the three stages (F _(2,27)_ = 0.09, p = 0.967) (**Figure 6**). Total daily rumination duration was 209.3 ± 27 min (14.5% of the daily 24h), 203.5 (14.1% of 24h) ± 28 min, and 220.8 ± 30 min (15,3% of 24 h) in stages 1, 2, and 3, respectively. These findings indicate that, unlike changes in feeding mode (1 food distribution, 4 food distributions, *ad libtum* or deprivation) explored in experiment 1 which influence the total daily rumination duration, changes in the timing of food distribution (experiment 2) do not affect this parameter.

## 4 Discussion

Several studies have examined rumination and its rhythm, with most reporting the existence of a rhythmic rumination profile (Bell and Lawn, 1957b; Gordon and McAllister, 1970; Oshiro, 1985; Phillips, 2002; Lindgren, 2009; Schirmann et al., 2012; Soriani et al., 2013; M’Hani et al., 2024). The present work provides experimental evidence of a circadian origin of the rumination rhythm. In the case of a single daily food distribution (stage 1), the rumination rhythm closely resembled that observed under the same dietary conditions under natural environment, with an acrophase occurring towards the end of the subjective night. This similarity to natural conditions (i.e. presence of LD cycles) could be explained by the fact that feeding time may synchronise rhythms either throughout the SCN or independently of the SCN. It has been demonstrated in several laboratory rodents that feeding can influence multiple rhythmic physiological and behavioural functions by acting on peripheral clocks, particularly in the liver and the intestine (Damiola et al., 2000; Stephan, 2001; Hara et al., 2001; Stokkan et al., 2001; Hoogerwerf et al., 2007). Indeed, several studies in rodents have clearly demonstrated that the gastrointestinal tract particularly the small intestine, harbors an intrinsic circadian clock, characterized by the daily rhythmic expression of clock genes (*Clock*, *Bmal1*, *Per*, *Cry* and ReverbA). This clock is entrained by the timing of nutrient availability, independently of the suprachiasmatic nuclei (SCN) (Hoogerwerf et al., 2007; Bron and Furness, 2009; Balakrishnan et al., 2010). The intestinal clock has been demonstrated to regulate a variety of functions related to digestion, including motility, cell proliferation, and nutrient absorption (Pan and Hussain, 2009; Balakrishnan et al., 2010; Ma et al., 2023). Therefore, it is hypothesized that the gastro-intestinal clock may also play a role in synchronizing the rumination rhythm with feeding time, independent of the suprachiasmatic nucleus (SCN). In addition, in case of the absence of LD cycles in goat, food intake has been shown to entrain the onset of locomotor activity (Giannetto et al., 2010). In sheep, even in the presence of LD cycles, food availability restricted to the dark phase was able to entrain the locomotor activity rhythm, shifting it from a predominantly diurnal pattern to a predominantly nocturnal one (Piccione et al., 2007). During stage 1 of both experiments, it was noted that the duration of the rumination phase (i.e., the interval between the onset and offset of rumination) appeared significantly longer compared to that observed under natural conditions. M’Hani et al. (2024) reported that the phase of the rumination rhythm is influenced by photoperiod. It was found to be more extended in winter, when the dark phase of the light-dark cycle is longer. Under constant darkness (DD) conditions, animals tended to distribute their rumination over a prolonged period. This was particularly evident given the single daily feeding, which forced the animals to feed at a fixed time and devoted the remainder of the day to rumination. Despite the flattened profile observed under these conditions, the rhythm remained robust, with a clear acrophase occurring at the end of the subjective night.

The recorded rumination duration of 291.6 ± 41 minutes in stage 1 was close to that observed in the same breed of goats under outdoor natural environmental conditions (winter and summer: 280 ± 37 and 281 ± 36 min, respectively) (M’Hani et al., 2024). Based on studies in cattle, thes findings suggest that rumination time is stable within the same breed and positively correlated with breed body weight (Aikman et al., 2008; Prendiville et al., 2010; Pereira and Heins, 2019). When comparing with the goat breed used in our study, longer rumination durations have been reported in other goat breeds under the same regime of a single daily food distribution. These breeds include, the English breed (464 min/day) (Bell and Lawn, 1957b), the Bligon (438.4 min/day) (Budisatria et al., 2014), and Saanen (329.8 min/day) (Oshiro et al., 1992), all of which have higher average body weights than the Moroccan Black goat (Hossaini-Hilali and Benlamlih, 1995).

When the feeding schedule changed to four distributions per day (00h, 06h, 12h and 18h) in stage 2, the rumination rhythm persisted with a period very close to, and statistically not different from, 24 hours. Since four daily food distributions are theoretically expected to eliminate food intake as a temporal cue (Refinetti, 2006; El Allali et al., 2013), this raises an important question of why the rumination rhythm did not exhibit a clear free-running pattern during this stage and instead appear on the ruminogram (mean of 4 animals) as divided into 4 blocks of rumination episodes? To answer this question, we should consider several physiological particularities of ruminants. In these species, the greatest feeding activity typically occurs immediately after food is distributed (King et al., 2016) and importantly, rumination does not occur during or immediately after feeding, due to the existence of an inhibition period (lag period) after eating and before the onset of rumination (Pearce, 1965b). Consequently, the rumination rhythm was probably interrupted at each new feeding distribution, preventing the expression of a clear free-running pattern.

In stage 3, food was provided *ad libitum*, thereby eliminating food distribution as a potential confounding factor affecting the rumination rhythm. Consequently, the animals were under strict constant conditions without any temporal perception. It was only under these constant conditions that evidence supporting the circadian origin of the rumination rhythm appeared. Indeed, the rumination rhythm persisted with a period significantly longer than 24.0 h (Tau=24.9 h), which is consistent with the diurnal nature of the goat (Piccione et al., 2008; Farsi et al., 2018). This observation aligns with Aschoff’s third rule, which states that under DD, the free-running period of diurnal animals tends to be longer than 24 hours (Aschoff, 1981; Beaulé, 2008). The phase delay of the rumination rhythm was confirmed by a significant shift in the acrophase from 06 h 43 ± 65 min in stage 2 to 12 h 54 ± 49 min in stage 3. This phase shift is clearly visible in the double-plotted ruminograms, in which the rhythm exhibits progressive daily phase delay. This typical free-running profile closely resembles the previously observed free-running pattern of locomotor activity in goats (Farsi et al., 2020a). This finding strongly suggests the involvement of the central master clock in modulating the circadian rhythm of rumination.

During stage 3 of experiment 1, the total duration of rumination was significantly decreased compared to stage 1 of the same experiment. This decrease was expected given the *ad libitum* feeding schedule. Under this feeding mode, animals spend more time eating and chewing, both of which are negatively correlated with rumination duration (Dado and Allen, 1994; Oshiro, 1996; Schirmann et al., 2012).

When the animals were deprived of food for four days (Stage 4), their rumination rhythm persisted. This supports the conclusion that rumination and its rhythmicity are not generated by food intake (Welch and Smith, 1968; Oshiro et al., 1992; M’Hani et al., 2024). The acrophase continued to shift, occurring at 17:19 ± 64 min and the period (23.2 h) became shorter than in phase 3 (24.9h). This significant change in the period reflects a disruption of the rumination rhythm, once again highlighting the influence of feeding behaviour on this rhythm.

Results of experiment 2 first demonstrated that shifting the time of food distribution does not affect the mean daily total rumination duration. This finding is consistent with observations in other ruminants. Indeed, in sheep, a 4-hour delay in food distribution did not influence daily rumination time (Gordon, 1958b). Similarly, in dairy cows, a 3.5-hour shift in food distribution, although it slightly affected dry matter intake, did not alter total daily rumination duration (King et al., 2016). However, studies investigating the effect of shifting food distribution time on rumination duration provided no clear conclusions regarding its impact on the rumination rhythm. In this context, the present study is the first to demonstrate that a 12-h shift in the time of food distribution under constant condition (DD and Cta) leads to an immediate parallel shift of rumination rhythm. The return in stage 3 to the initial time of food distribution (10:00 h) induced a return to the initial phase (phase delay) of the rumination rhythm. Given the strong evidence that has been presented for the potential circadian origin of the rumination rhythm, the question is raised of by which mechanism a shift in food delivery time is able to entrain the rumination rhythm. Is this due to an effect on the SCN or other circadian clocks and may some direct behavioural (i.e. masking) responses participate in the phase shift of rumination rhythm after acute change in the time of food distribution. Upon examining the results, we observed that the phase shift in the rumination rhythm occurred immediately following the shift in food distribution time, whereas entrainment of the master circadian clock to feeding time is known to occur progressively over several days, as demonstrated in mice, rats, goat, and dromedary camel (Castillo et al., 2004; Holmes and Mistlberger, 2000; Marchant and Mistlberger, 1996; Caldelas et al., 2005; Cambras et al., 1993; Farsi et al., 2020a, Farsi et al., 2020b, Farsi et al., 2022). This immediate shift also resembles the free-running profile observed in Experiment 1 (**Figure 2**). The nature of this phase shift suggests that the change in feeding time exerts a direct effect on the rhythm independently of the SCN. However, this does not allow to exclude the possibility of a direct effect of feeding time on the SCN. In fact, zeitgebers are known to act on the period and phase of the rhythm (Refinetti, 2010a), while masking cues strengthen or weaken the rhythm and affect generally the amplitude and the shape (Refinetti, 2010b; Hanneman, 2001; Kelly, 2007). The results demonstrate that shifting the timing of food distribution was able to invert the phase of the rhythm while maintaining its synchronization to a 24-hour period. However, the amplitude remained unaffected throughout the experiment, and the robustness was not altered between stages 1 and 2. This provides evidence supporting a potential effect on the SCN.

## 5 Conclusion

This study demonstrates that the rumination rhythm is a real circadian rhythm and that it is synchronized by the timing of food availability. Further investigations are required to elucidate the full neurobiological mechanisms underlying the circadian control of rumination.

## Data Availability

The original contributions presented in the study are included in the article/supplementary material. Further inquiries can be directed to the corresponding author.
